# (3*E*,5*Z*)-Octadien-2-ol is an Aggregation-Sex Pheromone Produced by Males of the Queensland Longhorned Beetle, *Acalolepta aesthetica *(Olliff 1890)

**DOI:** 10.1007/s10886-026-01735-1

**Published:** 2026-07-22

**Authors:** Matthew D. Ginzel, Jorden Zarders, Kyeongnam Kim, Ellen J. Dunkle, Kylle Roy, Dong H. Cha, Jocelyn G. Millar

**Affiliations:** 1 Department of Entomology, Purdue University, West Lafayette, IN 47907 USA; 2 Department of Forestry and Natural Resources, Purdue University, West Lafayette, IN 47907 USA; 3 Daniel K. Inouye U.S. Pacific Basin Agricultural Research Center, USDA Agricultural Research Service, Hilo, HI 96720 USA; 4 Department of Plant and Environmental Protection Sciences, University of Hawaii at Manoa, Hilo, HI 96720 USA; 5 Oak Ridge Institute for Science and Education, Oak Ridge, TN 37831 USA; 6 Research Corporation of the University of Hawaiʻi, Pacific Cooperative Studies Unit, Hilo, HI 96720 USA; 7 Forest Health Protection, Pacific Southwest Region, USDA Forest Service, Hilo, HI 96720 USA; 8 Department of Entomology, University of California, Riverside, CA 92521 USA

**Keywords:** Invasive species, Semiochemical, Pheromone chemistry, Chirality, Enantiomer

## Abstract

We describe the identification, synthesis, and field testing of an aggregation-sex pheromone produced by male *Acalolepta aesthetica* (Olliff 1890) (Coleoptera: Cerambycidae: Lamiinae), a cerambycid beetle native to Australia that has recently become established in Hawai‘i. Comparing extracts of headspace volatiles from both sexes revealed that males emitted two sex-specific volatiles, (3*E*,5*Z*)-octadien-2-ol and (3*E*,5*Z*)-octadien-2-one. Both compounds were synthesized and their bioactivity was tested in field bioassays. Only (3*E*,5*Z*)-octadien-2-ol was attractive to beetles of both sexes in these trials, and the corresponding ketone appeared to antagonize attraction when combined with the alcohol. In a dose-response study, traps baited with all doses of (3*E*,5*Z*)-octadien-2-ol except 33.3 mg captured more beetles than solvent-control traps, with no significant differences among the effective doses. In a subsequent field trial, beetle capture did not differ between 100 mg of a racemic blend and the insect-produced (*R*)*-*enantiomer of this compound, indicating that the unnatural (*S*)-enantiomer is not antagonistic. No other cerambycid species were captured during our field trials. The structural motif of (3*E*,5*Z*)-octadien-2-ol is unique among pheromones identified from lamiines to date. Together, our findings provide a foundation for developing pheromone-based tools to detect, delimit and potentially manage *A. aesthetica*.

**Supplementary Information:**

The online version contains supplementary material available at https://doi.org/10.1007/s10886-026-01735-1.

## Introduction

*Acalolepta aesthetica* (Olliff 1890) (Cerambycidae: Lamiinae), commonly known as the Queensland longhorned beetle, is native to eastern Queensland and New South Wales, Australia (Secretariat 2023), and has recently become established in the State of Hawai‘i, USA. Since its initial discovery in 2009 in the Puna District of Hawaiʻi Island (Matsunaga and Chun 2018), it has spread throughout North and South Hilo and Hāmākua Districts (Sofaer et al. 2025). This species has a broad host range in Hawai‘i, including an estimated 13 host species representing eight orders and 11 families (Hawaiʻi Department of Agriculture 2020). Documented hosts of *A. aesthetica* include ecologically, economically, and culturally important plants such as ʻulu (*Artocarpus altilis* Parkinson (*Fosberg*), kukui (*Aleurites moluccanus* (L.)Willd.), *Citrus* spp., cacao (*Theobroma cacao* L.), alaheʻe (*Pysdrax odorata* (G. Forst.) A.C. Sm. & S.P. Darwin; S. Chun, personal communication), and avocado (*Persea americana* Mill.; Hawaiʻi Department of Agriculture 2020). Continued expansion of *A. aesthetica* within Hawaiʻi not only threatens the health and productivity of its forest ecosystems and agricultural commodities, but also poses a biosecurity risk to other Hawaiian islands, the continental United States, and other Pacific regions.

Effective management of invasive woodborers like *A. aesthetica* depends on early detection and delimitation of incipient populations, and rapid responses to control and, if possible, eradicate the beetles before they cause devastating ecological and economic losses (Williams et al. 2023). Detection protocols for *A. aesthetica* currently rely on visual surveys for physical signs of infestations (e.g., larval frass, branch dieback, or adult exit holes), which are labor-intensive approaches that typically detect infestations only after beetle populations are well established (Matsunaga and Chun 2020). In contrast, pheromone-based approaches have proven effective for detecting many cerambycid species, particularly for those in the subfamily Lamiinae, where adult males produce pheromones that attract both sexes (Millar and Hanks 2017). Identification of an attractant pheromone for *A. aesthetica* has the potential to provide more sensitive and selective approaches to reducing its impact, and to limit its expansion within Hawai‘i and incursions into other parts of the world.

Many lamiine species share conserved pheromone motifs (Millar and Hanks 2017). For example, hydroxyethers such as monochamol are widely used within the tribe Monochamini, and terpenoid-derived compounds such as fuscumol, fuscumol acetate, and related degradation products of geranylacetone are broadly attractive to multiple taxa (Millar and Hanks 2017). Congeners of *A. aesthetica* were reported to be attracted to monochamol in field trials conducted in China (Wickham et al. 2014) and to several common cerambycine pheromones in Australia (Hayes et al. 2016). However, attempts to capture *A. aesthetica* in Hawai‘i using known cerambycid pheromones, including monochamol, fuscumol, fuscumol acetate, and geranylacetone, were not successful (Collignon et al. 2019). This lack of response to known pheromones of lamiine species suggested that *A. aesthetica* might produce novel semiochemicals for mate location, rather than using one of the known structural motifs typical of many lamiine pheromones.

Here, we report the identification of (3*E*,5*Z*)-octadien-2-ol as the principal and possibly only male-produced attractant pheromone of *A. aesthetica*. Specifically, we describe the isolation and structural identification of two male-produced compounds, their synthesis, and the results of field assays testing their bioactivity. We also conducted a dose–response field study of the major component to determine an effective lure loading for field applications, and analyzed its enantiomeric composition. We confirmed that the beetles produce the (*R*)-enantiomer of (3*E*,5*Z*)-octadien-2-ol and evaluated its biological activity compared to that of a racemic mixture under field conditions. Collectively, these results provide a foundation for the development of pheromone-based surveillance and management strategies for *A. aesthetica.*

## Methods and Materials

### Collection and Analysis of Pheromone Candidates

Adult beetles were hand-collected from kukui trees at dusk from May – July 2022 at the Kamehameha Schools Hawaiʻi Campus in Keaau, HI, USA. Beetles were then transported to the Daniel K. Inouye U.S. Pacific Agricultural Research Center (Hilo, HI) and maintained outdoors in individual screen cages that were sheltered from rain. Beetles were provided fresh kukui leaves and stems for food and a moistened dental wick for supplemental water.

We reasoned that *A. aesthetica* may require host plant to emit pheromones, so we collected headspace volatiles from male and female beetles on a kukui stem with 2–3 leaves, that were held individually in 2.4 L glass chambers (Chemglass Life Sciences, Vineland, NJ, USA) that contained a piece of aluminum mesh as a perch for the beetles. Volatiles were collected using a single ORBO™-32 standard charcoal tube filter (Supelco, Bellefonte, PA, USA) modified with silanized glass wool (Supelco, Bellefonte, PA, USA), connected to the outlet of each chamber with a screw cap fitted with a Teflon™ ferrule. Air entering each chamber was scrubbed through a charcoal filter and drawn through the system at 0.6 L/min by a vacuum pump. Beetles were aerated continuously for 72 h under ambient outdoor conditions and collection filters were replaced each day. A total of five males and three females were aerated no more than four times each. Volatiles were eluted from ORBO filters with 600 µL of methylene chloride (Supelco, Bellefonte, PA, USA).

To determine whether the host plant had any influence on pheromone production, adult males were also aerated in the absence of host material. Thus, males were starved for at least 24 h to allow them to empty their alimentary tract prior to collecting volatiles from beetles without host plant material (i.e., no kukui) using methods described above. In total, eight males were aerated no more than three times each. These aerations occurred in July 2024, and beetles were provided by the USDA Agricultural Research Service, National Germplasm Repository in Hilo, HI.

Because *A. aesthetica* is in the subfamily Lamiinae, for which all pheromones identified to date have been male-produced aggregation-sex pheromones, we looked for male-specific compounds in volatiles collected from live insects. Samples were initially analyzed using gas chromatography-mass spectrometry (GC-MS). An autosampler injected 1 µL of sample into an Agilent GC-MS (Agilent Technologies, Santa Clara, CA, USA; Model numbers 7890B and 5977 A, respectively) equipped with a polar column (DB-WAXETR; 30 m × 0.25 mm × 0.25 μm film thickness; Agilent), with a flow rate of 1 ml/min of helium carrier gas under splitless injection mode with an inlet temperature of 250 °C. Oven temperature was initially held at 40 °C for 5 min and then ramped at 15 °C/min to 250 °C and held for 5 min. Mass spectra were obtained by electron impact ionization (EI) at 70 eV.

Aeration extracts of live beetles, prepared in Hawai‘i, were shipped to University of California Riverside, Riverside, CA, USA (UC Riverside), where they were analyzed by GC-MS on an Agilent 7820 A GC fitted with a DB-5 column (30 m × 0.25 mm ID, 0.5 μm film thickness; Agilent), interfaced to an Agilent 5977E mass selective detector. Helium was used as carrier gas, and the oven was programmed from 40 °C for 5 min, then 10 °C/min to 280 °C, hold for 10 min. Transfer line and injector temperatures were 250 °C. Injections were made in splitless mode and mass spectra were obtained in EI mode (70 eV), scanning a mass range of *m/z* 41–500. Extracts were also analyzed on a Hewlett-Packard (H-P) 6890 GC equipped with a DB-17 column (30 m × 0.25 mm ID, 0.5 μm film thickness; J&W Scientific, Folsom, CA, USA) and interfaced to an H-P 5973 mass selective detector, using run parameters analogous to those described above.

To determine the enantiomeric composition of analytes, an aliquot of an extract was reduced by stirring with ~ 1 mm^3^ of 5% Pd on carbon under H_2_ atmosphere for 30 min. The slurry was then filtered through a plug of celite, and the reduced extract was analyzed by GC-MS as described above, to confirm that the reduction had been successful. The reduced extract was then analyzed on a Cyclodex-B chiral stationary phase column (30 m × 0.25 mm ID, 0.25 μm film thickness; J&W Scientific), with a temperature program of 50 °C/1 min, then 3 °C/min to 220 °C, hold for 10 min. Standard samples of racemic and (*R*)-2-octanol (Oakwood Chemical, Estill, SC, USA) were run under identical conditions.

## Synthesis of Pheromone Candidates

Tetrahydrofuran was purified by distillation from sodium-benzophenone ketyl. Reactions using air- or water-sensitive reagents were carried out under Ar atmosphere in oven-dried glassware. Unless otherwise stated, solutions were dried over anhydrous Na_2_SO_4_ and concentrated by rotary evaporation under partial vacuum. Vacuum flash chromatography was performed with 230–400 mesh silica gel. Yields have not been optimized.

## Preparation of all 4 isomers of 3,5-octadien-2-ol

### (3*E*,5*E*)-Octadien-2-ol

A dry flask under Ar was charged with 15 ml dry THF and (2*E*,4*E*)-heptadienal **1** (1.1 g, 10 mmol). The flask was cooled to -78 °C and methylmagnesium bromide (3 M in diethyl ether, 4 ml, 12 mmol) was added by syringe pump over 30 min. The mixture was stirred 1 h at -78 °C, then quenched by dropwise addition of saturated aq. NH_4_Cl. The resulting mixture was warmed to room temp, diluted with water, and extracted with ether. The ether layer was washed with brine, dried, and purified by Kugelrohr distillation (bp ~ 65 °C at 3.5 mm Hg) yielding (3*E*,5*E*)-octadien-2-ol [(**3*E***,**5*E*)-2**] as a colorless oil (1.05 g, 85%). EIMS, *m/z* (abundance): 126 (31), 111 (15), 109 (10), 108 (65), 97 (52), 95 (11), 91 (68), 84 (9), 83 (14), 81 (20), 79 (82), 78 (17), 77 (81), 71 (13), 69 (33), 68 (63), 67 (44), 66 (13), 65 (20), 58 (12), 57 (11), 55 (48), 53 (22), 51 (14), 45 (9), 43 (100), 41 (36).

### Blends of 3,5-octadien-2-ols

Methyl lactate **3** (5.2 g, 50 mmol) was taken up in 200 ml CH_2_Cl_2_, and the solution was cooled in an ice bath. Imidazole (3.74 g, 55 mmol) was added in one portion, followed by *t*-butyldimethylsilyl chloride (9.06 g, 60 mmol). The mixture was warmed to room temp and stirred 2 h, then quenched with 100 ml water. After stirring 5 min, the layers were separated, and the aqueous phase was extracted again with CH_2_Cl_2_. The combined organic layers were washed with dilute aq. NaHCO_3_ and brine, dried, and concentrated. The residue was purified by Kugelrohr distillation, taking a forerun (bp < 40 °C at 4 mm Hg), then the bulk of the protected alcohol **4** (bp ~ 60 °C at 4 mm Hg, 10.77 g, 99%).

A portion of the distillate (2.18 g, 10 mmol) was taken up in dry CH_2_Cl_2_ in a flask under Ar, the solution was cooled to − 78 °C, and diisobutylaluminum hydride (DIBALH, 1 M in toluene, 13 ml, 13 mmol) was added dropwise over 30 min. The mixture was stirred 2 h, then quenched at − 78 °C by dropwise addition of 10 ml of a saturated aqueous solution of sodium potassium tartrate tetrahydrate. The mixture was warmed to room temp, stirred 2 h, then filtered through a plug of celite, rinsing with CH_2_Cl_2_. The layers were separated and the organic layer was dried and concentrated under partial vacuum to remove the CH_2_Cl_2_. The resulting solution (~ 15 ml) of the crude aldehyde **5** in toluene was used in the next step without further purification.

A solution of (*Z*)-2-pentenyl bromide (8.94 g, 60 mmol) and triphenylphosphine (15.72 g, 60 mmol) in 150 ml toluene was allowed to stand at room temp for 7 d. The resulting white crystals were filtered off, rinsed with toluene, and dried under vacuum, yielding (*Z*)-2-pentenyltriphenylphosphonium bromide **(*Z*)-6.**

Under Ar, butyllithium (2.5 M in hexanes, 1 ml, 2.5 mmol) was added to a slurry of **(*Z*)-6** (1.03 g, 2.5 mmol) in ~ 25 ml dry THF at 0 °C, and the bright red mixture was stirred 30 min. A 1.5 ml aliquot of the solution if crude aldehyde **5** (nominal 1 mmol) was then added dropwise over 30 min. The mixture was warmed to room temp and stirred 1 h, then quenched with saturated aq. NH_4_Cl, diluted with water, and extracted with hexane. The hexane layer was washed, dried, and concentrated, and the residue was taken up in 5 ml hexane to precipitate most of the triphenylphosphine oxide side product. A 1 ml aliquot of the liquid portion was concentrated, then stirred overnight in 1.5 ml of 1 M tetrabutylammonium fluoride in THF. The mixture was then diluted with water, extracted with hexane, and the hexane layer was washed with water and brine, then dried and concentrated. The residue was purified by vacuum flash chromatography, eluting with 15% EtOAc in hexane, yielding 55 mg of the 3,5-dien-2-ol **2** as a ~ 1:1 mixture of (3*Z*,5*Z*)- and (3*E*,5*Z*)-isomers.

The (3*E*,5*E*)- and (3*Z*,5*E*)-isomer pair were made in analogous fashion by using (*E*)-2-pentenyltriphenylphosphonium bromide **(*E*)-6** as a reactant. **(*E*)-6** was prepared as described above for **(Z)-6**, substituting (*E*)-2-pentenyl bromide for (*Z*)-2-pentenyl bromide.

### (3*E*,5*Z*)-Octadien-2-ol

A solution of 3-butyn-2-ol **7** (26.75 g, 382 mmol) and 0.5 g p-toluenesulphonic acid in 250 ml dry diethyl ether was cooled in an icebath, and ethyl vinyl ether (48 ml, 500 mmol) was added by syringe pump over 1 h. The mixture was warmed to room temp, and stirred until the starting alcohol had been entirely consumed. The cloudy pale brown mixture was then washed twice with saturated aq. NaHCO_3_, once with brine, dried, and then concentrated under reduced pressure. The slightly viscous opaque residue was then Kugelrohr distilled (bp ~ 45 °C, 7.5 mm Hg), yielding the protected alkynol **8** as a clear, colorless liquid (48.54 g, 89.5%) as a ~ 2:1 mixture of diastereomers. EIMS of major isomer, *m/z* (abundance): 141 (M^+^−1, 1), 127 (28), 97 (44), 89 (5), 75 (43), 73 (83), 69 (9), 61 (10), 55 (26), 53 (95), 47 (19), 45 (100), 43 (30).

A dry 3-neck flask under Ar was charged with ~ 500 ml dry THF and protected alkynol **8** (35.5 g, 250 mmol) and cooled in an ice bath. Butyllithium (2.5 M in hexanes, 105 ml, 263 mmol) was added by syringe pump over 1 h, and the resulting solution was stirred 1 h at 0 °C. The mixture was then cooled to ~ − 15 °C in an ice-salt bath, and paraformaldehyde (15 g, 500 mmol, dried under vacuum over P_2_O_5_ for 24 h) was added in one portion, giving a milky yellow slurry. The mixture was warmed to room temp and stirred 2 h, giving a clear pale brown solution. The mixture was cooled in an ice bath again and quenched with sat. aq. NH_4_Cl solution. The mixture was stirred for 10 min, then extracted twice with hexane (300 ml, then 100 ml). The combined organic extracts were washed with brine, dried, concentrated, and the resulting orange oil was Kugelrohr distilled, taking a forerun with bp < 65 °C at 4.5 mm Hg, followed by the desired product **9** (bp ~ 60–70 °C at 0.05 mm Hg) as a colorless oil (35.8 g, 83%, ~ 2:1 mixture of diastereomers). EIMS of major isomer, *m/z* (abundance): 154 (M^+^-18, 12), 139 (3), 125 (24), 109 (64), 97 (9), 93 (13), 91(10), 86 (6), 81 (16), 79 (19), 77 (15), 73 (100), 67 (23), 55 (11), 45 (83), 43 (20).

A dry 1 L 3-neck flask was charged with ~ 500 ml dry THF, cooled in an ice-salt bath, and under a positive flow of Ar, LiAlH_4_ (8.36 g, 220 mmol) was added in 4 portions over 5 min. Alkynol **9** (35.8 g, 208 mmol) was then added by syringe pump over 1 h, and the resulting slurry was stirred overnight at 0 °C in an insulated bath. The mixture was then quenched by sequential syringe pump addition of water (8.8 ml over 1 h), 20% aq. NaOH (6.6 ml over 20 min), and water (30.8 ml in one portion). The resulting slurry was stirred 1 h, then filtered with suction, rinsing twice with THF. The resulting clear solution was concentrated and purified by Kugelrohr distillation (bp ~ 65 °C at 0.05 mm Hg) yielding alkenol **10** as a clear, colorless oil (19.53 g, 54%, ~ 2:1 mixture of diastereomers; (*Z*)-isomers not detected). EIMS of major isomer, *m/z* (abundance): 159 (M^+^-15, 1), 129 (1), 99 (28), 97 (10), 85 (40), 73 (100), 67 (20), 57 (20), 55 (14), 45 (81), 43 (29).

A 1 L 3-neck flask was fitted with a thermometer, and charged with ~ 500 ml CH_2_Cl_2_, Dess-Martin periodinane (53 g, 125 mmol) and powdered NaHCO_3_ (34 g, 400 mmol). Alkenol **10** (15 g, 86 mmol) was added over 30 min with a syringe pump, keeping the mixture at or slightly below room temp with a cold water bath. When the addition was complete, the cooling bath was removed and the mixture was stirred 1 h. The reaction was then worked up by removing most of the CH_2_Cl_2_ by rotary evaporation. The residue was taken up in 250 ml pentane, resulting in a voluminous precipitate, which was removed by suction filtration, rinsing the filter cake twice with pentane. A 1:1 mixture of 10% sodium thiosulphate: saturated NaHCO_3_ (400 ml) was then added in portions (Caution! Foams! ), and the mixture was stirred 1 h. The layers were separated, and the pentane layer was washed with a further 150 ml of the 1:1 mixture of 10% sodium thiosulphate: saturated NaHCO_3_. The layers were separated, the organic layer was washed with brine, dried, concentrated, and purified by Kugelrohr distillation (bp ~ 75 °C at 0.05 mm Hg) yielding the aldehyde **11** as a colorless oil (9.66 g, 65%, ~ 2:1 mixture of diastereomers). EIMS of major isomer, *m/z* (abundance): 157 (M^+^-15, 4), 127 (7), 100 (11), 83 (100), 73 (82), 55 (49), 45 (70), 43 (17).

An oven-dried 1 L 3-neck flask was charged with propyltriphenylphosphonium bromide (30.28 g, 75 mmol) and ~ 500 ml dry THF. Sodium hexamethyldisilazide (40 ml, 2 M in THF) was added by syringe pump over 1 h, giving a red-orange slurry. The mixture was stirred 2 h, then aldehyde **11** (10 g, 58 mmol) in 20 ml THF was added by syringe pump over 1 h. The pale orange slurry was stirred 1 h, then quenched by dropwise addition of 10 ml saturated ag. NH_4_Cl. The THF was removed by rotary evaporation, and the residue was triturated with 250 ml pentane. The pentane slurry was filtered with suction, rinsing the cake twice with pentane. The organic solution was washed with water and brine, dried, and concentrated. The residue was purified by Kugelrohr distillation, taking a forerun with bp < 40 °C at 0.05 mm Hg, then the bulk of the product at ~ 75 °C at 0.03 mm Hg, yielding the protected dienol **12** (10.36 g) as a mixture of isomers. This was taken up in 100 ml THF, cooled to 0 °C, and 1 M aq. HCl was added (10 ml). The mixture was stirred overnight at 0 °C, then saturated aq. NaHCO_3_ (25 ml) and diethyl ether (100 ml) were added. The organic layer was washed with brine, dried, concentrated, and purified by Kugelrohr distillation (bp ~ 80 °C at 5 mm Hg), yielding dienol **2** as an 85:15 mixture of (3*E*,5*Z*)- and (3*E*,5*E*)-isomers (6.3 g, 86% from aldehyde **11**).

A portion of the 85:15 mixture of isomers (1 g, 7.9 mmol) was taken up in 25 ml CH_2_Cl_2_ at room temp, and tetracyanoethylene (0.2 g, 1.6 mmol) was added in portions over 10 min. The resulting dark purple-brown solution was stirred at room temp overnight, yielding a dirty yellow solution. The mixture was concentrated, then triturated with 10 ml hexane. The hexane soluble portion was purified by vacuum flash chromatography, eluting with a stepwise gradient of 10%, 16.6%, and 25% EtOAc in hexane. The fractions containing dienol **(3*E***,**5*Z*)-2** were combined and Kugelrohr distilled, yielding 0.43 g in 99% isomeric purity. EIMS, *m/z* (abundance): 126 (23), 111 (12), 109 (4), 108 (17), 97 (35), 95 (4), 91 (16), 84 (4), 83 (11), 81 (12), 79 (22), 78 (4), 77 (23), 71 (10), 69 (25), 68 (52), 67 (33), 66 (5), 65 (9), 58 (11), 57 (6), 55 (41), 53 (15), 51 (8), 45 (10), 43 (100). ^1^H NMR (400 MHz, CDCl_3_): δ 6.51 (dd, 1H, J = 15.19, 11.06 Hz), 5.96 (overlapped dd, 1H, J ~ 10.9 Hz), 5.73 (dd, 1H, J = 15.19, 6.49 Hz), 5.47 (dt, 1H, J = 10.72, 7.57 Hz), 4.39 (m, 1H), 2.22 (qdd, 2 H, J ~ 7.5, 7.5, 1.35 Hz), 1.57 (1H, broad), 1.32 (d, 3 H, J = 6.39 Hz), 1.02 (t, 3 H, J = 7.53 Hz). ^13^C NMR: 136.77, 134.68, 127.04, 124.90, 68.77, 23.35, 21.08, 14.20 ppm.

### Preparation of (3*E*,5*Z*)-Octadien-2-one

An oven-dried flask was charged with Dess-Martin periodinane, (3.4 g, 8 mmol), powdered NaHCO_3_ (2.1 g, 25 mmol), and 50 CH_2_Cl_2_, and the mixture was cooled in an ice bath. A solution of (3*E*,5*Z*)-octadien-2-ol (0.75 g, 6 mmol) in 5 ml CH_2_Cl_2_ was then added by syringe pump over 30 min. The mixture was stirred 2 h, then concentrated by rotary evaporation. The residue was stirred with 100 ml 10% aq. sodium thiosulphate solution for 1 h, then extracted with ether. The ether solution was dried and concentrated, then purified by vacuum flash chromatography, with a step gradient of 10 and 20% ether in pentane. The product was further purified by Kugelrohr distillation (0.47 g, 63%, bp ~ 65 °C at 4 mm Hg). EIMS, *m/z* (abundance): 124 (34), 109 (18), 95 (100), 82 (9), 81 (56), 79 (35), 77 (10), 67 (7), 66 (7), 65 (9), 55 (9), 53 (23), 51 (9), 43 (41), 41 (16).

### Preparation of (2*R*,3*E*,5*Z*)-Octadien-2-ol

Bromine (12.8 ml, 250 mmol) was added by syringe pump over 2 h to a solution of (*E*)-2-pentenoic acid **12** (25.1 g, 251 mmol) in 100 ml CH_2_Cl_2_, and the solution was stirred overnight. The solution was concentrated, and the resulting viscous orange oil was taken up in 50 ml dimethylformamide. The solution was added over 45 min by syringe pump to a slurry of NaHCO_3_ (36 g, 430 mmol) in 120 ml DMF maintained at 70 °C, with the system under partial vacuum (100 mm Hg), collecting the crude bromoalkene in a dry ice cooled trap. As the dibromoacid was added, the reaction mixture foamed and turned dark brown. Once the addition was complete, the mixture was stirred an additional 30 min at 70 °C, then the vacuum was increased to 60 mm Hg. The vacuum was then released, and the cold trap contents (clear liquid) were warmed to room temp and diluted with 50 ml pentane. The solution was washed with water and brine, and the pentane was removed by distillation through a jacketed Vigreux column, warming the solution in an oil bath at 50 °C at atmospheric pressure. (*Z*)-1-Bromo-1-butene **13** was obtained as a colorless liquid (22.6 g, 67%), which was stored in a − 70 °C freezer until used.

(*R*)-3-Butyn-2-ol **(*R*)-7** (Arctom, Westlake Village, CA) was protected as the 1-ethoxyethyl ether, **(*R*)-8**, as described for the racemic compound.

A dry 500 ml 3-neck flask was charged with bistriphenylphosphinepalladium dichloride (0.94 g, 2 mmol) and CuI (1.4 g, 7.3 mmol), flushed thoroughly with Ar, and maintained under Ar throughout. Dry THF (150 ml) was added, followed by the protected alkynol **(*R*)-8** (9.94 g, 75 mmol) and (*Z*)-1-bromo-1-butene **13** (16.8 g, 124 mmol). Diisopropylamine (18.75 ml, 133 mmol) was then added over 1 h by syringe pump, and the reaction mixture turned black. The mixture was stirred at room temp for 6 d, then diluted with hexane and filtered with suction. The filtrate was washed twice with saturated NH_4_Cl, then brine, and concentrated. The residue was taken up in hexane and purified by vacuum flash chromatography, eluting with 6% EtOAc in hexane. The fractions containing the enyne product were combined and purified further by Kugelrohr distillation, yielding 8.8 g (60%) of the protected enynol **(*R*)-14** as a clear oil (bp ~ 75 °C at 0.5 mm Hg), which was taken up in 100 ml of a 9:1 mixture of THF and 1 M aq. HCl. The solution was stirred for 4.5 h at room temp, then diluted with 100 ml pentane, and extracted twice with 25 ml saturated aq. NaHCO_3_, then brine. After concentration, the residue was purified by Kugelrohr distillation (bp ~ 75–80 °C at 5.5 mm Hg), yielding enynol **(*R*)-15** (4.98 g, 53% from alkyne **(*R*)-8**). ^1^H NMR (400 MHz, CDCl_3_): δ 5.92 (dt, 1H, J = 10.8, 7.3 Hz), 5.46 (br d, 1H, J ~ 10.8 Hz), 4.69 (br quart, 1H, J ~ 6.2 Hz), 2.31 (quint d, 2 H, J = 7.5, 1.4 Hz), 2.05 (br s, 1H), 1.50 (d, 3 H, J = 6.6 Hz), 1.03 (t, 3 H, J = 7.6 Hz). ^13^C NMR: 146.04, 107.67, 95.10, 80.70, 58.90, 24.48, 23.63, 13.31 ppm.

A dry 500 ml flask under Ar was charged with 75 ml dry THF, cooled in an ice bath, and LiAlH_4_ (2.66 g, 20 mmol) was added in portions over 5 min, followed by addition of (*R*)-enynol **(*R*)-15** (4.25 g, 34 mmol) in 10 ml THF with a syringe pump over 30 min. The cooling bath was then removed and the mixture was stirred overnight, then quenched by sequential dropwise addition of water (2.67 ml), 20% aq. NaOH (2 ml), and water (9.38 ml). The resulting slurry was stirred 30 min, then filtered with suction, rinsing with ether. The filtrate was dried, then concentrated and Kugelrohr distilled, yielding **(2*R***,**3*E***,**5*Z*)-2** (3.63 g, 85%, bp ~ 80 °C at 7 mm Hg, contaminated with ~ 8% of the (2*R*,3*E*,5*E*)-isomer). The spectral data matched those of the racemic compound.

## Field Bioassays for Candidate Pheromone Components

All field bioassays were conducted within Liko Nā Pilina project plots at Keaukaha Military Reservation (KMR; 19°42’15” N, -155°2’40” W) in Hilo on the Island of Hawaiʻi. Liko Nā Pilina is a hybrid forest ecosystem restoration project established in 2011 that aims to design restoration treatments for wet lowland forests that improve invasion resistance while including agroforestry plants for sustainability (Ostertag et al. 2020). We chose plots that contained signs of *A. aesthetica* damage (e.g., exit holes and frass) and its preferred hosts including kukui, ʻulu, avocado, and alaheʻe. The elevation is 30 m.a.s.l. with a 750–1500 year old ʻāʻā lava flow and average rainfall of 3,347 mm/yr and mean annual temperature of 27.7 °C (Giambelluca et al. 2013, 2014). KMR encompasses a highly degraded wet lowland forest dominated by ʻōhiʻa trees (*Metrosideros polymorpha*) that are affected by the fungal disease complex rapid ʻōhiʻa death, and the mid-canopy and understory are heavily invaded by invasive plants.

We conducted the following three field bioassays to test for the attraction of *A. aesthetica* to (3*E*,5*Z*)-octadien-2-ol and (3*E*,5*Z*)-octadien-2-one: 

**Experiment 1** tested for attraction of the beetle to the major component, (3*E*,5*Z*)-octadien-2-ol, alone and when combined with (3*E*,5*Z*)-octadien-2-one. The experiment consisted of five blocks of three traps that were baited with 40 mg of (3*E*,5*Z*)-octadien-2-ol in 1 ml isopropyl alcohol (IPA), 40 mg of (3*E*,5*Z*)-octadien-2-ol + 8 mg of (3*E*,5*Z*)-octadien-2-one in 1 mL IPA, or with an IPA control. This experiment was conducted from 31 May to 21 June 2024 (four weeks).

**Experiment 2** tested the response of *A.* *aesthetica* to four doses of (3*E*,5*Z*)-octadien-2-ol from 3 July to 30 July 2025 at KMR (five weeks). Three blocks of traps were baited with lures that contained either 3.3, 10, 33.3 or 100 mg of synthetic pheromone in 1 mL IPA, and 1 mL IPA as a control.  

**Experiment 3** compared the attraction to racemic and chiral (3*E*,5*Z*)-octadien-2-ol and consisted of five blocks of three traps that were baited with either racemic (3*E*,5*Z*)-octadien-2-ol (100 mg) or (2*R*,3*E*,5*Z*)-octadien-2-ol (50 mg) in IPA, or a control lure of IPA, and was deployed from 28 August to 18 September 2025 (four weeks). The 100 mg dose was selected for subsequent field trials because it was clearly active and provided a conservative lure loading for maximizing detection sensitivity, despite not differing significantly in bioactivity from the other effective doses.

We used flight intercept traps (black corrugated plastic, cross-vane, Alpha Scents, Portland, OR, USA) coated with Fluon PTFE (Northern Specialty Chemicals, Dudley, MA, USA; Graham et al. 2010) to enhance trapping efficiency. Pheromone lures were zip-lock polyethylene sachets (Bagettes model 14770, 5.1 × 7.6 cm; Cousin, Largo, FL, USA) loaded with synthetic compounds in 1 mL IPA or 1 mL IPA controls. 

For all experiments, traps were spaced at least 10 m apart and with 30 m between transects. Traps were hung from tree branches using the manufacturer provided metal hangars, approximately ~1 m from the ground. For experiments 2 and 3, traps were modified to accept XL screw-top plastic collection jars that were filled with ~200 mL of a 1:1 mixture of LowTox® propylene-glycol antifreeze (Prestone®, Danbury, Connecticut, USA) and water as a killing agent. Captured beetles were collected from traps on a weekly basis, and all captured beetles were sexed based on the length of their antennae relative to body size (Collignon et al. 2019). 

### Data Analysis

For all three field experiments, replicates were defined by trap collections conducted across transects and collection dates (i.e., replication over space and time). Treatments were rotated among trap positions each collection period to control for potential location effects. Because trap-catch data violated the assumptions of parametric analyses, treatment effects were analyzed using rank-based nonparametric methods (Sokal and Rohlf 1995). Raw trap-catch data were converted to ranks (Zar 1999), and overall treatment effects were evaluated using Cochran–Mantel–Haenszel statistics based on rank scores (PROC FREQ, option CMH2; SAS Institute 2011). To facilitate interpretation of treatment differences, ranked data were subsequently analyzed using the Ryan–Einot–Gabriel–Welsch Q (REGWQ) multiple-comparison procedure (SAS Institute 2011).

## Results

### Collection, Analysis, and Identification of Beetle-Produced Compounds

**Fig. 1:**
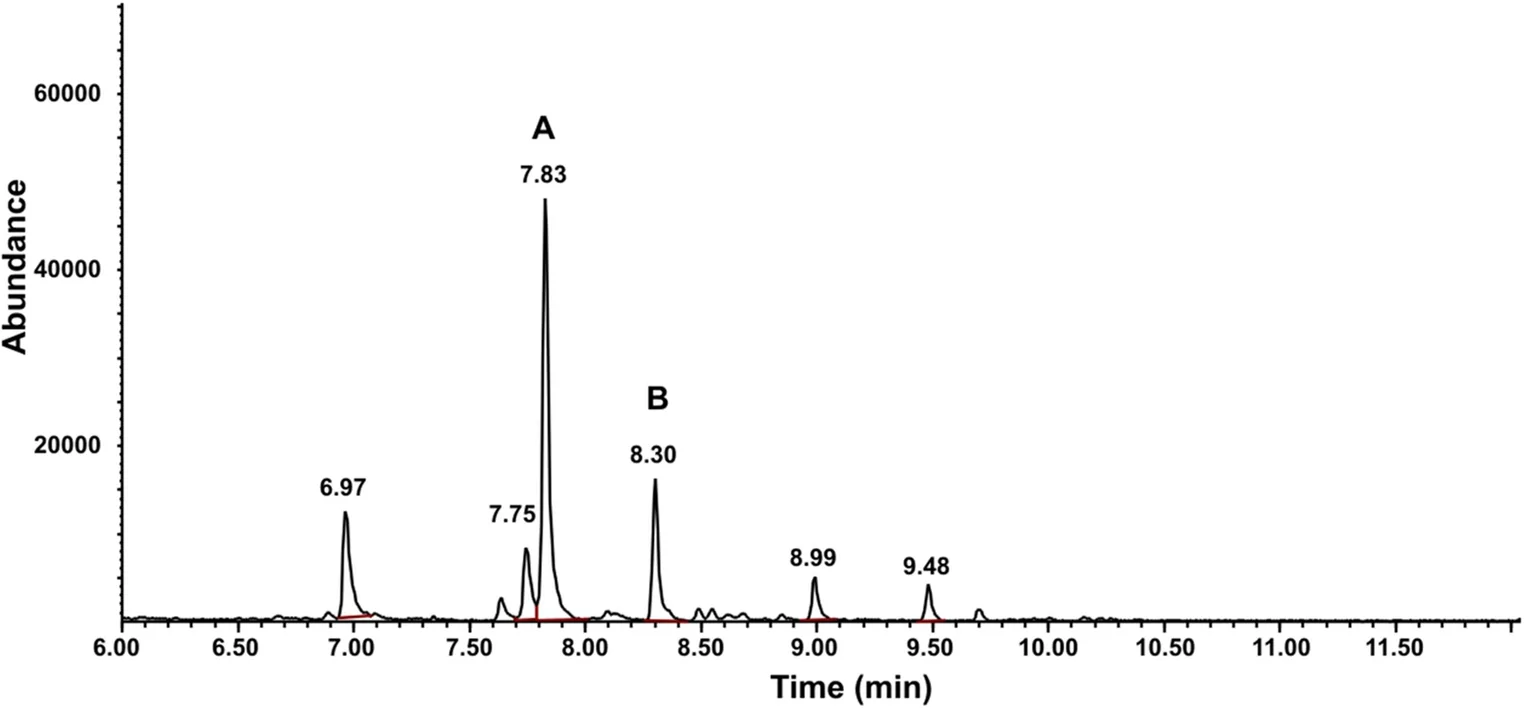
Representative total ion chromatogram of an extract of volatiles from male *Acalolepta* *aesthetica* maintained on kukui twigs. Peak identifications: 6.97 min, (*E*)-3-octen-2-one; 7.64 min, *p*-cymene; 7.75 min, β-phellandrene; **7.83 min, (3*E*,5*Z***)**-octadien-2-ol (Peak A)**; 8.13 min, unknown; **8.30 min, (3*E*,5*Z***)**-octadien-2-one (Peak B)**; 8.99 min, phenylethanol; 9.48 min, unidentified oxygenated monoterpene. Compounds in bold were insect-produced

**Fig. 2:**
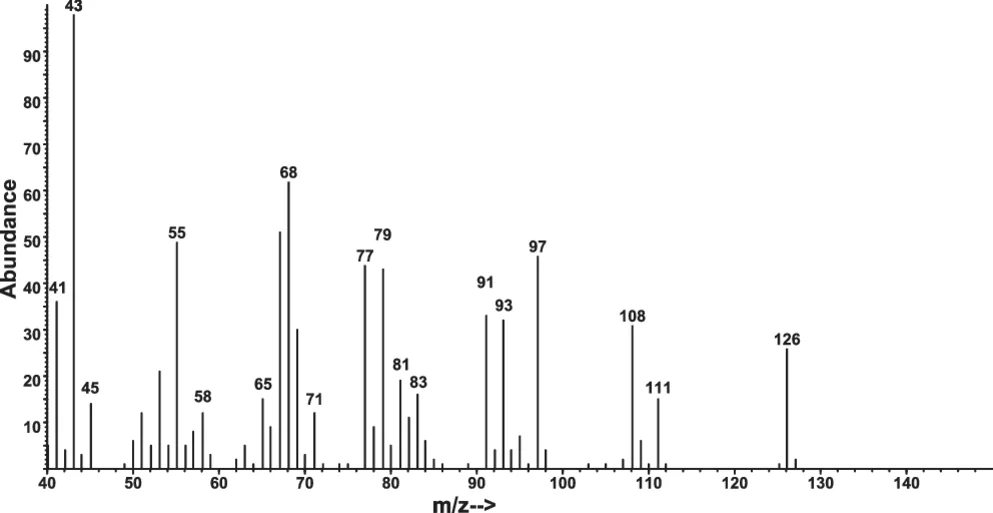
EI mass spectrum of beetle-produced (3*E*,5*Z*)-octadien-2-ol

**Fig. 3:**
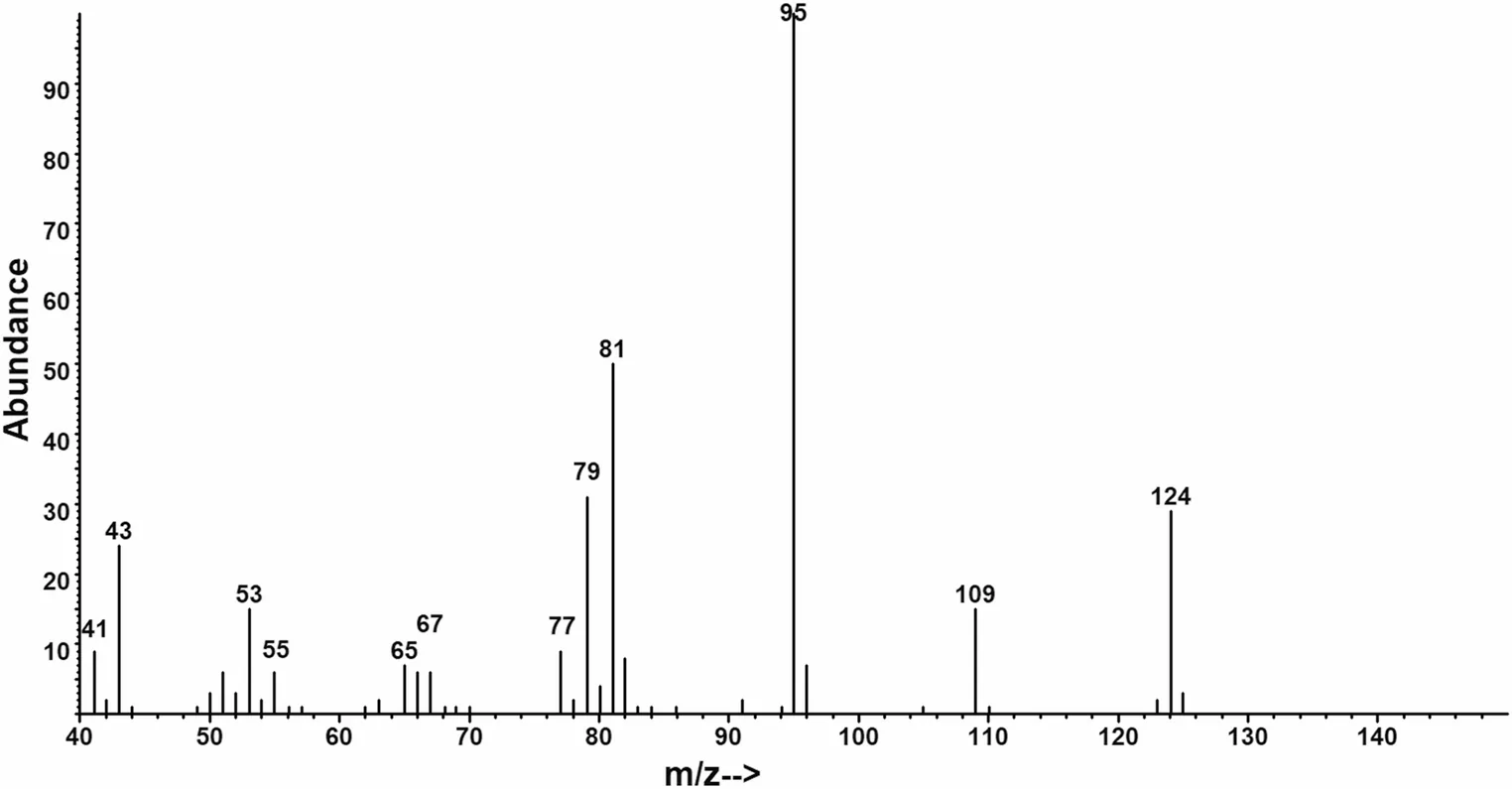
EI mass spectrum of beetle-produced (3*E*,5*Z*)-octadien-2-one

Aeration extracts of male beetles consistently contained two peaks (Fig. 1) which were not present in extracts of females (see Supplementary Fig. S1). The later-eluting peak **B** (retention time 8.31 min), with a likely molecular ion at *m/z* 124 (Fig. 3), was tentatively identified as a 3,5-octadien-2-one isomer via a reasonable mass spectral match with a NIST database spectrum (74%). There were no plausible database matches for the first-eluting peak **A** (Fig. 2), but the fact that it had a likely molecular ion at *m/z* 126 suggested that it might be either the corresponding diene alcohol, or an octenone. The latter possibility was discounted because the mass spectrum was substantially different than that of a 3-octen-2-one standard. 

**Scheme 1:**
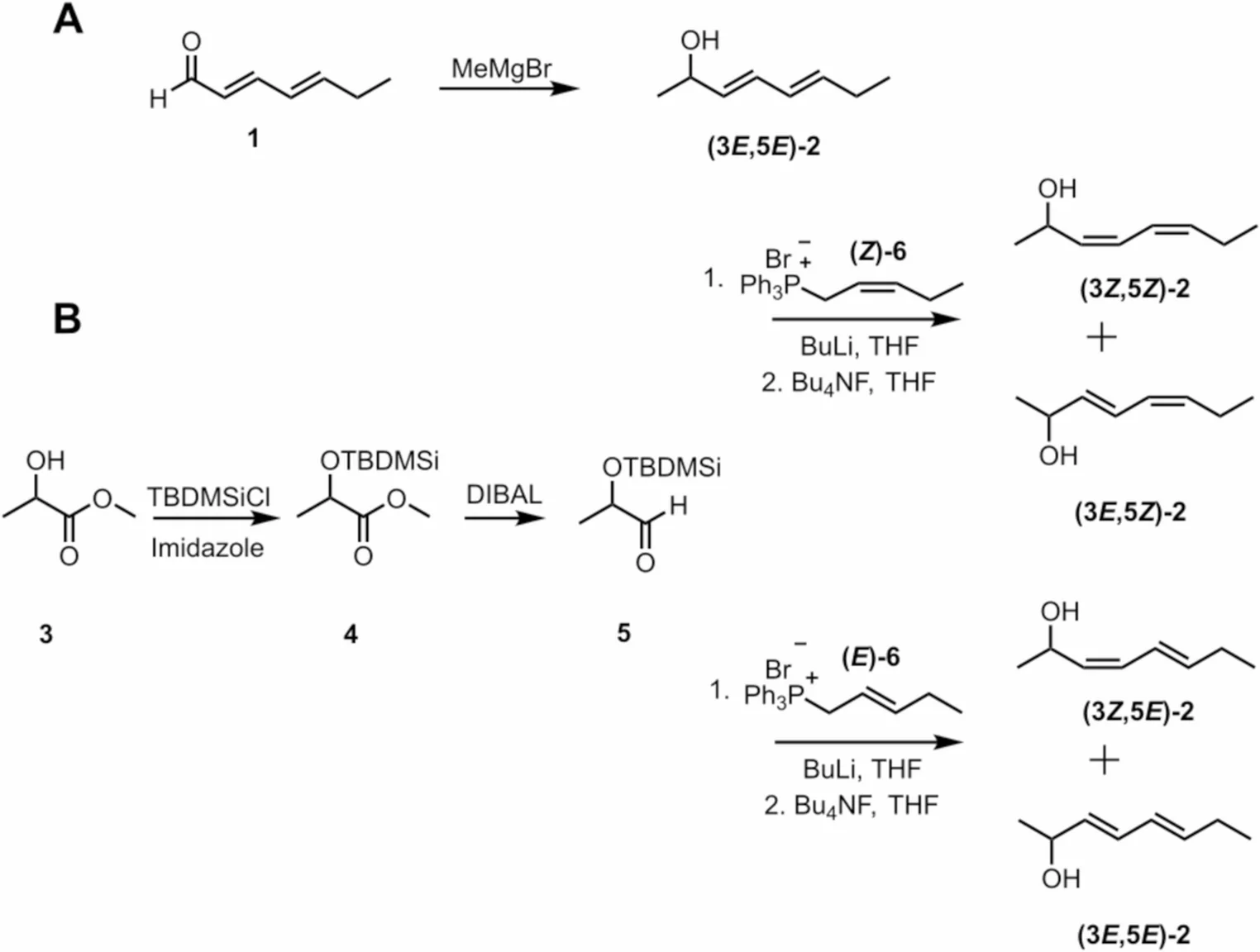
(**A**) Stereoselective synthesis of (3*E*,5*E*)-octadien-2-ol; (**B**) Synthesis of all four isomers of 3,5-octadien-2-ol, as two blends of two isomers each

The basic dienol and dienone structures were verified by reduction of an aliquot of an extract with hydrogen and 5% Pd/C catalyst, which produced 2-octanol and 2-octanone respectively. In addition, the fact that the retention times of the insect-produced dienol and dienone were markedly longer than those of 2-octanol and 2-octanone on a nonpolar DB-5 GC column indicated that the compounds were conjugated. Furthermore, the insect-produced dienone eluted later than the dienol, suggesting that the ketone functionality in the former compound was conjugated to the C = C double bonds, indicating a 3,5-placement of the double bonds in both compounds. This was verified by synthesis of a standard of (3*E*,5*E*)-octadien-2-ol by reaction of (2*E*,4*E*)-heptadienal **1** with methylmagnesium bromide (Scheme 1A). The resulting (3*E*,5*E*)-octadien-2-ol [**(3*E***,**5*E*)-2**] eluted slightly later than the insect-produced compound, but it contained a small percentage of an isomer which matched both the retention time and the mass spectrum of the insect-produced compound. Because it was very unlikely that the synthesis from (2*E*,4*E*)-heptadienal **1** would produce even a trace of the (3*Z*,5*Z*)-product, we reasoned that the insect compound had to be either the (3*E*,5*Z*)- or (3*Z*,5*E*)-isomer, with the former being favored because (*Z*)-2-alkenals are very unstable and rapidly isomerize to the (*E*). The (3*E*,5*Z*)-configuration was proven by synthesis of two standard mixtures (Scheme 1B), by Wittig reaction of 2-(*t*-butyldimethylsilyloxy)propanal **5** with either (*E*)- or (*Z*)-2-pentenyltriphenylphosphonium bromides **6**, yielding mixtures of the (3*E*,5*E*)- and (3*Z*,5*E*)-isomers (retention times 7.92 and 7.67 min) and the (3*E*,5*Z*)- and (3*Z*,5*Z*)-isomers (ret times 7.83 and 7.78 min) of **2** respectively. Similarly, the insect-produced dienone was confirmed as (3*E*,5*Z*)-octadien-2-one by matching its retention time with a standard generated by oxidation of the appropriate mixture of alcohols. 

Finally, to determine which enantiomer of the alcohol component was produced by the beetles, the extracts and appropriate standards were analyzed on a chiral stationary phase Cyclodex-B GC column. Racemic (3*E*,5*Z*)-octadien-2-ol was not resolved on this column, but the reduced form, 2-octanol, did resolve. Analysis of a commercial sample of (*R*)-2-octanol versus racemic 2-octanol, and a coinjection of the two, determined that the (*R*)-enantiomer eluted first. Analysis of the reduced insect extract (see above), alone and as a coinjection with the racemic standard, then determined that the insect-produced alcohol had the (*R*)-configuration.

### Large-scale synthesis of putative pheromone components

**Scheme 2:**
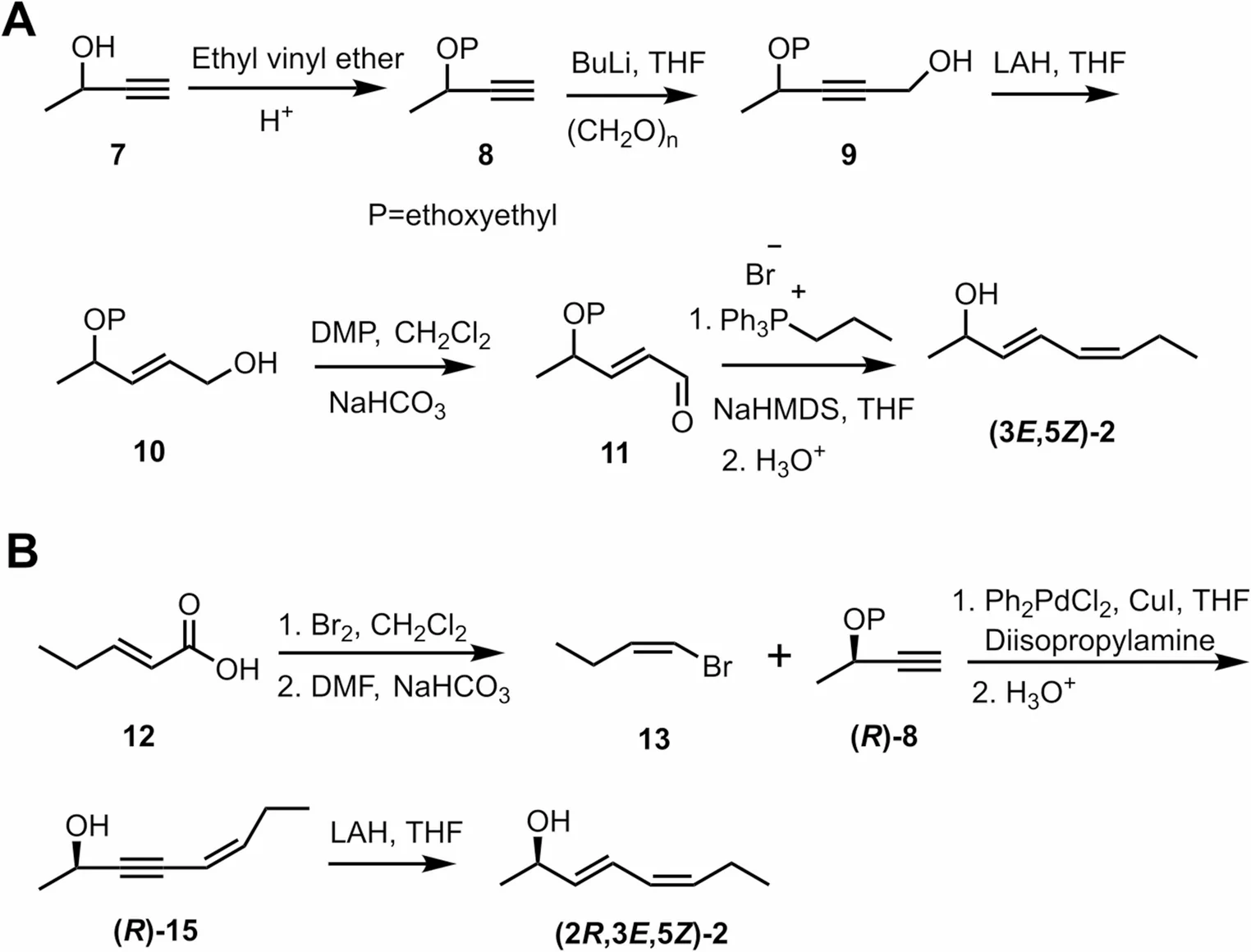
Multigram syntheses of (A) racemic (3*E*,5*Z*)-octadien-2-ol; B) (2*R*,3*E*,5*Z*)-octadien-2-ol

Our first attempt at synthesizing the basic skeleton of (3*E*,5*Z*)-octadien-2-ol by Pd-catalyzed coupling of 3-butyn-2-ol and (*Z*)-1-bromo-1-butene resulted in a very poor yield (< 10%), and so to provide material quickly for field trials, we developed a longer but more straightforward synthesis that did not rely on a metal-catalyzed coupling (Scheme 2A). Thus, the alcohol function of 3-butyn-1-ol **7** was protected as the ethoxyethyl ether **8**, which was then deprotonated with butyllithium and reacted with paraformaldhyde, giving alcohol **9**. This was stereoselectively reduced to the (*E*)-alkenol **10** with LiAlH_4_. Oxidation of the alcohol with Dess-Martin periodinane yielded the conjugated aldehyde **11**, which was then stereoselectively coupled with propyltriphenylphosphonium bromide in THF, with sodium hexamethyldisilazide (NaHMDS) as base, giving the protected dienol **12**. Acid-catalyzed hydrolysis of the ethoxyethyl protecting group then gave racemic (3*E*,5*Z*)-octadienol **2**. 

To synthesize the insect-produced enantiomer, i.e., (2*R*,3*E*,5*Z*)-octadien-2-ol, we reinvestigated the shorter, Pd-catalyzed route mentioned above, using freshly purified intermediates and reagents (Scheme 2B). This time, the key Pd-catalyzed coupling of ethoxyethyl-protected (*R*)-3-butyn-2-ol **(*R*)-8** with (*Z*)-1-bromo-1-butene **13** (readily prepared from inexpensive (*E*)-2-pentenoic acid **12;** Hickman et al. 2011) proceeded in reasonable yield, albeit slowly (6 d), giving the protected enynol **(*R*)-14**. Removal of the protecting group gave enynol **(*R*)-15**, which was stereoselectively reduced with LiAlH_4_ to the desired (2*R*,3*E*,5*Z*)-octadien-2-ol, **(2*R***,**3*E***,**5*E*)-2**.

### Field Bioassays of Candidate Pheromone Compounds

**Fig. 4:**
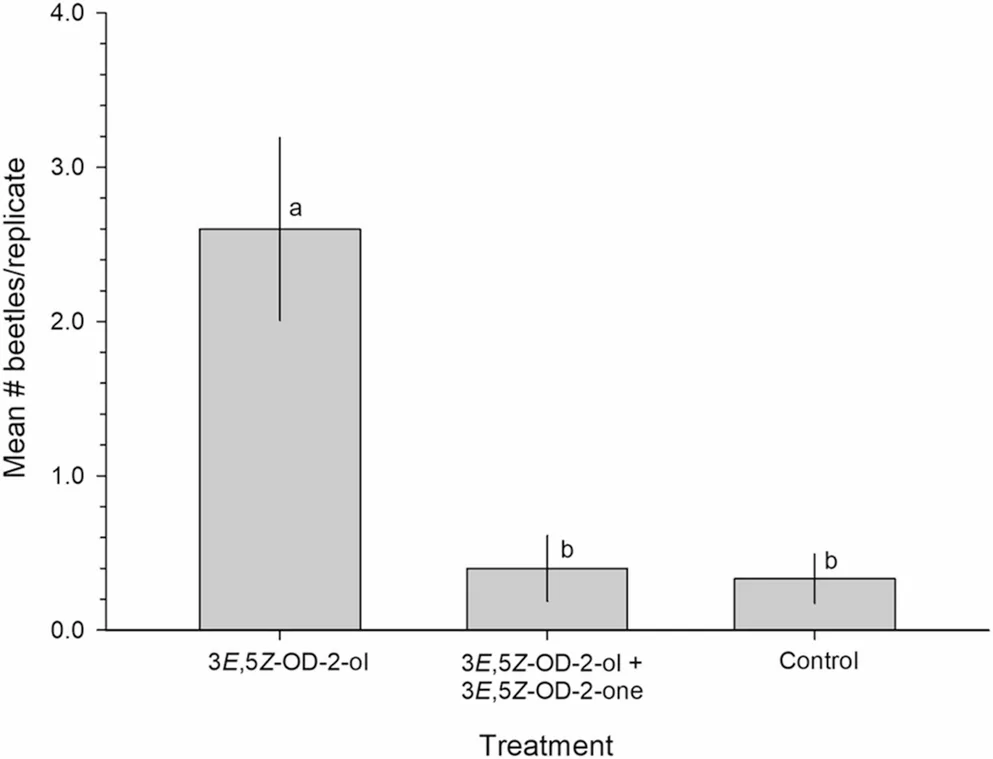
Mean (±SE) number of adult *Acalolepta aesthetica* captured per replicate (n = 20) in response to (3*E*,5*Z*)-octadien-2-ol, (3*E*,5*Z*)-octadien-2-ol + (3*E*,5*Z*)-octadien-2-one, and control treatments (Experiment 1). Chemical abbreviations: 3*E*,5*Z*-OD-2-ol = (3*E*,5*Z*)-octadien-2-ol; 3*E*,5*Z*-OD-2-one = (3*E*,5*Z*)-octadien-2-one. Means with different letters are significantly different (REGWQ test, P<0.05)

Across the three field bioassays, *A. aesthetica* adults were consistently attracted to (3*E*,5*Z*)-octadien-2-ol. In the first experiment comparing (3*E*,5*Z*)-octadien-2-ol versus the blend of (3*E*,5*Z*)-octadien-2-ol and (3*E*,5*Z*)-octadien-2-one, we captured a total of 21 males and 29 females. Trap captures differed significantly among treatments (CMH test based on rank scores: χ² = 14.38, df = 2, *P* = 0.0008). Traps baited with (3*E*,5*Z*)-octadien-2-ol captured significantly more *A. aesthetica* (mean ± SE = 2.60 ± 0.59) than traps baited with the blend of (3*E*,5*Z*)-octadien-2-ol and (3*E*,5*Z*)-octadien-2-one (mean ± SE = 0.40 ± 0.21) or the control (mean ± SE = 0.33 ± 0.16), indicating that attraction to the alcohol was antagonized by the presence of the ketone (Fig. 4). 

**Fig. 5:**
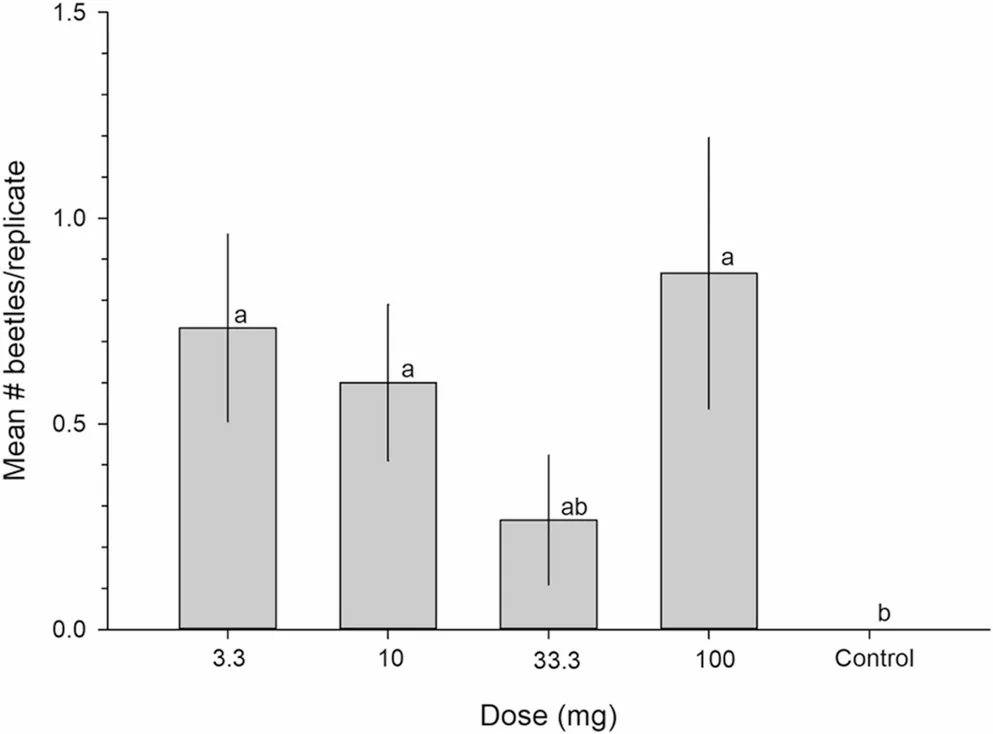
Mean (±SE) number of adult *Acalolepta aesthetica* captured per replicate (n = 15) testing the dose-response of beetles to (3*E*,5*Z*)-octadien-2-ol (Experiment 2). Means with different letters are significantly different (REGWQ test, P<0.05)

In the dose–response bioassay, a total of 11 males and 26 females were captured over the course of 5 wk. Trap captures differed significantly among treatments (CMH test based on rank scores: χ² = 12.33, df = 4, *P* = 0.0151). Traps baited with all doses of (3*E*,5*Z*)-octadien-2-ol except 33.3 mg captured significantly more *A. aesthetica* than control traps, whereas catches did not differ significantly among the effective doses. Mean (± SE) captures per trap were 0.00 ± 0.00, 0.73 ± 0.23, 0.60 ± 0.19, 0.27 ± 0.15, and 0.87 ± 0.34 beetles for the control, 3.3, 10, 33.3, and 100 mg treatments, respectively. No monotonic dose–response relationship was evident across the range of doses tested. Although mean captures were highest at the 100 mg dose, captures did not differ significantly among the active pheromone treatments (Fig. 5). 

**Fig. 6:**
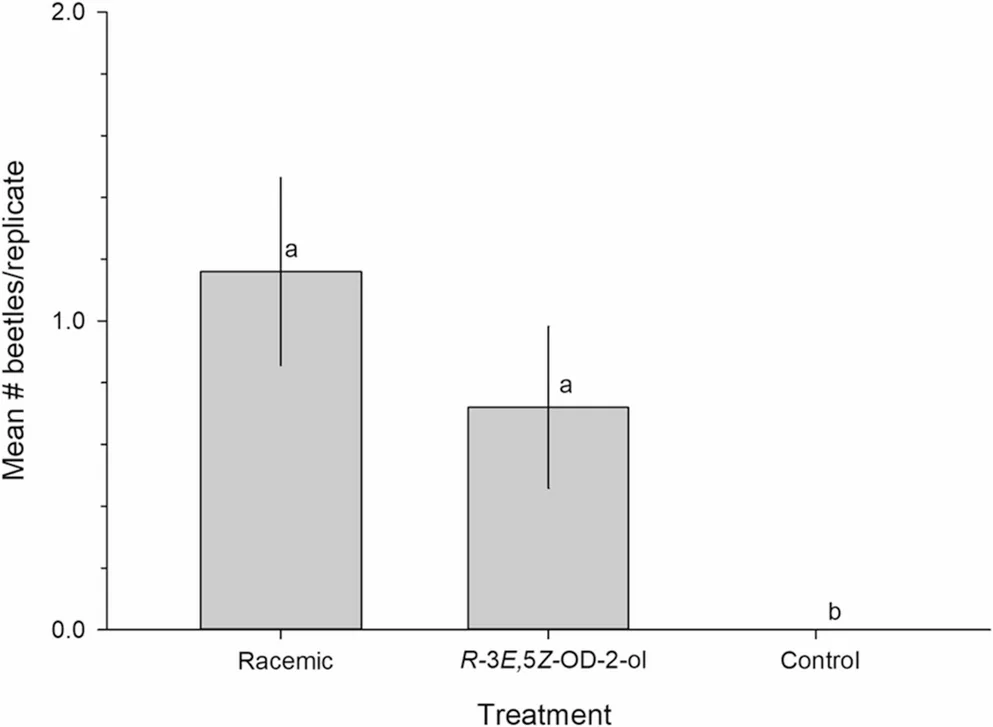
Mean (±SE) number of adult *Acalolepta aesthetica* captured per replicate (n = 20) in response to a racemic blend and the (*R*)-enantiomer of (3*E*,5*Z*)-octadien-2-ol (Experiment 3). Chemical abbreviations: *R*-3*E*,5*Z*-OD-2-ol = (2*R*,3*E*,5*Z*)-octadien-2-ol. Means with different letters are significantly different (REGWQ test, P<0.05)

Trap captures differed significantly among treatments in the bioassay comparing racemic and (*R*)-(3*E*,5*Z*)-octadien-2-ol versus the control (CMH test based on rank scores: χ² = 14.79, df = 2, *P* = 0.0006) (Fig. 6). Traps baited with either racemic or (*R*)-(3*E*,5*Z*)-octadien-2-ol captured significantly more beetles than solvent-control traps, whereas captures did not differ between the two pheromone treatments, indicating that the (*S*)-enantiomer was not antagonistic. Mean (± SE) captures per trap were 1.16 ± 0.30, 0.72 ± 0.26, and 0.00 ± 0.00 beetles for racemic, chiral, and control treatments, respectively. In this experiment, we captured a total of 22 males and 25 females. No other cerambycid species were captured in any experiment.

## Discussion

Our findings provided compelling evidence that (3*E*,5*Z*)-octadien-2-ol is produced exclusively by male *A. aesthetica* and strongly suggests that it acts as an aggregation-sex pheromone. Most volatile pheromones identified in the subfamily Lamiinae to date fall into two broad structural motifs: hydroxyethers and terpenoid-derived compounds (e.g. Zhang et al. 2002; Pajares et al. 2010; Mitchell et al. 2011). Of the hydroxyethers, the pheromone of the Asian longhorned beetle, *Anoplophora glabripennis* (Motschulsky), consists of 4-(heptyloxy)butanol and 4-(heptyloxy)butanal (Zhang 2002), but elicits only weak attraction in field trials unless supplemented with host-derived volatiles or other pheromone components (e.g., (*E*,*E*)-farnesene; Crook et al. 2014, Nehme et al. 2010; Meng et al. 2014). Related hydroxyethers, including 2-(undecyloxy)ethanol (“monochamol”) originally identified from *Monochamus galloprovincialis* (Oliver) are widely conserved in the tribe Monochamini and have been used effectively in monitoring programs worldwide (see Pajares et al. 2010; Millar and Hanks 2017).

The second major pheromone motif includes geranylacetone, a product of sesquiterpenoid degradation, and its corresponding alcohol (*E*)-6,10-dimethylundeca-5,9-dien-2-ol (fuscumol), along with fuscumol acetate. These compounds attract adults of several lamiine species and are broadly regarded as “generic” pheromones within the subfamily (Silk et al. 2007; Mitchell et al. 2011; also see Millar and Hanks 2017). Despite their broad biological activity, the enantiomeric composition of naturally produced pheromones remains poorly resolved for most taxa, with field trials typically using racemic compounds (e.g., Hanks et al. 2012) or isomeric mixtures (Mitchell et al. 2011; Hanks et al. 2012; Wong et al. 2012). To date, only *Astyleiopus variegatus* (Haldeman) is known to produce a specific enantiomeric form, namely the (*S*)-enantiomers of both fuscumol and fuscumol acetate (Hughes et al. 2013). In addition, two lamiines native to eastern North America, *Astylopsis macula* (Say) and *Leptostylus transversus* Gyllenhal, use related terpene-derived compounds, such as (*S*)-6-methylhept-5-en-2-ol (sulcatol) and (*S*)-6-methylhept-5-en-2-one (sulcatone) as pheromone components, further illustrating the breadth of this structural motif (Meier et al. 2019).

In contrast, *A. aesthetica* did not respond to monochamol, fuscumol, fuscumol acetate, or geranylacetone, either singly or in blends (Collignon et al. 2019). This lack of response is notable given that the congener *Acalolepta formosana* (Breuning 1935) was reported to be attracted to monochamol under similar trapping conditions (Wickham et al. 2014), suggesting that monochamol might be a shared structural motif within the genus. However, (3*E*,5*Z*)-octadien-2-ol represents an entirely different and novel structural motif, as it is a C8 diunsaturated alcohol that lacks both the ether linkage typical of hydroxyether pheromones and the terpenoid backbone of fuscumol-related compounds.

The absence of cross attraction of any other cerambycids in our field assays and the novel structural motif of its pheromone suggests that *A. aesthetica* may use a unique chemical communication channel in Hawai‘i, consistent with the concept of “pheromone-free space” proposed by Millar and Hanks (2016, 2018). This hypothesis argues that shared pheromone structures among co-occurring species can lead to signal interference, reducing mate-finding efficiency and increasing Allee effects. Consequently, an exotic species such as *A. aesthetica* may be more likely to establish in a naïve environment such as Hawai‘i when its pheromone components are chemically distinct from those of related native species. Hawai’i has a remarkable radiation of over 145 native cerambycids, and little is known about their chemical communication (Gressitt 1972). It remains to be seen whether the pheromone structure of *A. aesthetica* is truly unique and species-specific, or whether other congeners in Australia or other parts of the world will be found to produce the same compound as additional pheromones are identified within the genus.

The potential minor component, (3*E*,5*Z*)-octadien-2-one, did not enhance attraction of *A. aesthetica* to (3*E*,5*Z*)-octadien-2-ol in our field bioassays, and somewhat surprisingly, significantly reduced attraction relative to the alcohol alone. Thus, its natural function is unclear. It may be a biosynthetic precursor to the alcohol structure, or perhaps function to prevent maladaptive cross attraction of sympatric species that use a related pheromone motif in its native range.

Our results also indicated that (3*E*,5*Z*)-octadien-2-ol is not host-derived, because it was not present in the aerations of females on kukui, and was produced by males in the absence of host plant material (see Supplementary Fig. S2). Male-specific production, together with the results of our field assays, supports its role as an aggregation-sex pheromone. Enantiomeric analysis further revealed that males produce only the (*R*)-enantiomer of (3*E*,5*Z*)-octadien-2-ol. However, our field bioassays demonstrated that racemic and (*R*)-enriched lures performed equivalently, suggesting that the non-natural (*S*)-enantiomer has no biological role or relevance to this species. The ability to use racemic material as a lure should reduce production costs, thus increasing the feasibility for large-scale manufacture and commercialization of pheromone-based detection and management tools for this species.

Finally, in a dose-response trial, our field assays revealed that traps baited with all doses other than 33.3 mg captured more beetles than those baited with the solvent control. However, trap captures were variable and inconsistent across most doses, possibly due to variability in pheromone release from the sachets or beetle detection thresholds, and declining emission rates between service intervals may have further affected trapping efficacy of lower doses.

The species-specificity and behavioral activity of (3*E*,5*Z*)-octadien-2-ol suggests that it has clear utility for targeted detection and monitoring of *A. aesthetica* populations. Such pheromone-based trapping has the potential to detect low-density populations and trigger timely management responses. This tool would not only support early detection and rapid response frameworks in Hawai‘i and other Pacific islands that it may invade, but also at high-risk points of entry in the continental United States and beyond. Future studies should be directed toward developing the operational efficacy of this pheromone tool, for example, by optimizing pheromone release devices and release rates, and refining trapping protocols such as trap type, placement, spacing, and seasonal timing. 

## Supplementary Information

Below is the link to the electronic supplementary material.

Supplementary Material 1

Supplementary Material 2

## Data Availability

No datasets were generated or analysed during the current study.
