# Supplementary material for: (3E,5Z)-Octadien-2-ol is an Aggregation-Sex Pheromone Produced by Males of the Queensland Longhorned Beetle, Acalolepta aesthetica (Olliff 1890)
Source: J Chem Ecol. 2026 Jul 22;52(4):66. doi: 10.1007/s10886-026-01735-1 (PMC13391651; doi:10.1007/s10886-026-01735-1)

**Article title:** “(3E,5Z)-Octadien-2-ol is an Aggregation-Sex Pheromone Produced by Males of the Queensland Longhorned Beetle, Acalolepta aesthetica (Olliff 1890)”

**Journal:** Journal of Chemical Ecology

**Authors:** Matthew D. Ginzel; Jorden Zarders; Kyeongnam Kim; Ellen J. Dunkle; Kylle Roy; Dong H. Cha; Jocelyn G. Millar

**Corresponding author affiliation:** Departments of Entomology and Forestry & Natural Resources, Purdue University, 901 W. State Street, West Lafayette, IN 47907 USA

**Corresponding author e-mail:** mginzel@purdue.edu

**Figure S2.** Representative chromatogram of a headspace aeration extract from a male Acalolepta aesthetica adult maintained without host plant material. Chromatograms were obtained on a DB-WAXETR capillary column. Peak A is (3E,5Z)-octadien-2-ol, and peak B is (3E,5Z)-octadien-2-one. The elution order of these compounds is reversed relative to that shown in Fig. 1 because these analyses were conducted on a DB-WAXETR column rather than a DB-17 column.
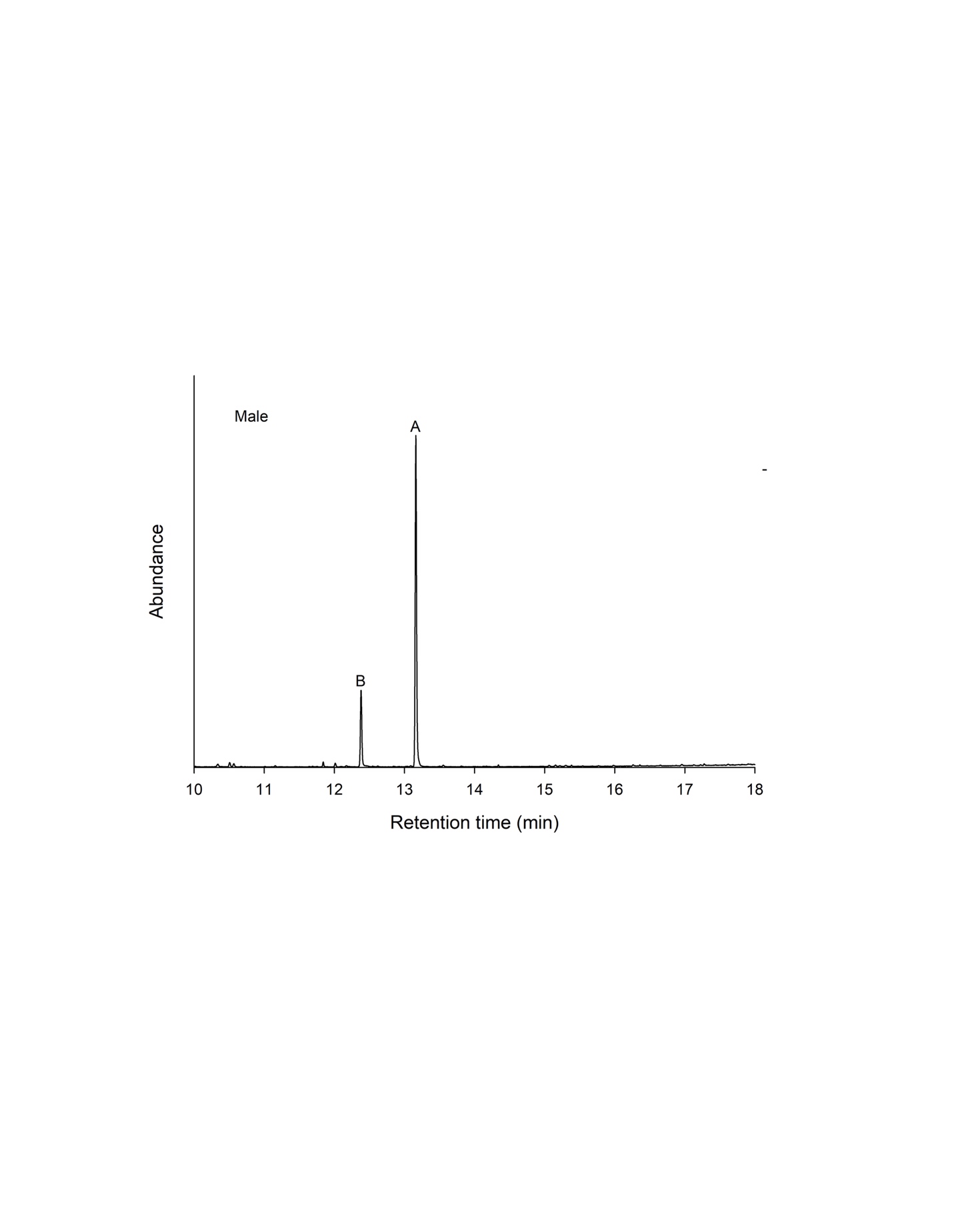

Supplement: Supplementary file 2 — Supplementary Material 2 [file 10886_2026_1735_MOESM2_ESM.docx]
